# Platelet-Rich Plasma (PRP) Mitigates Silver Nanoparticle (AgNP)-Induced Pulmonary Fibrosis via iNOS/CD68/CASP3/TWIST1 Regulation: An Experimental Study and Bioinformatics Analysis †

**DOI:** 10.3390/ijms26146782

**Published:** 2025-07-15

**Authors:** Shaimaa R. Abdelmohsen, Ranya M. Abdelgalil, Asmaa M. Elmaghraby, Amira M. Negm, Reham Hammad, Eleni K. Efthimiadou, Sara Seriah, Hekmat M. El Magdoub, Hemat Elariny, Islam Farrag, Nahla El Shenawy, Doaa Abdelrahaman, Hussain Almalki, Ahmed A. Askar, Marwa M. El-Mosely, Fatma El Zahraa Abd El Hakam, Nadia M. Hamdy

**Affiliations:** 1 Anatomy & Embryology Department, Faculty of Medicine (for Girls), Al-Azhar University, Nasr City 11754, Cairo, Egypt; 2 Histology Department, Faculty of Medicine (for Girls), Al-Azhar University, Nasr City 11754, Cairo, Egypt; 3 Physiology Department, Faculty of Medicine (for Girls), Al-Azhar University, Nasr City 11754, Cairo, Egypt; 4 Clinical Pathology Department, Faculty of Medicine (for Girls), Al-Azhar University, Nasr City 11754, Cairo, Egypt; 5 Institute of Nanoscience and Nanotechnology, National Center for Scientific Research “Demokritos”, 15341 Athens, Greece; 6 Laboratory of Inorganic Chemistry, Department of Chemistry, National and Kapodistrian University of Athens, 15772 Athens, Greece; 7 Department of Clinical, Pharmaceutical, and Biological Science, School of Life and Medical Sciences, University of Hertfordshire, Hatfield AL10 9AB, UK; 8 Biochemistry Department, Faculty of Pharmacy, Misr International University, Cairo 19648, Egypt; 9 Department of Pharmacology and Toxicology, College of Pharmacy, University of Hail (UoH), Hail 81481, Saudi Arabia; 10 Department of Pharmacology and Toxicology, Faculty of Pharmacy (for Girls), Al-Azhar University, Nasr City 11754, Cairo, Egypt; 11 Forensic Medicine and Clinical Toxicology Department, Faculty of Medicine (for Girls), Al-Azhar University, Nasr City 11754, Cairo, Egypt; 12 Department of Zoology and Entomology, Faculty of Science (for Girls), Al-Azhar University, Nasr City 11754, Cairo, Egypt; 13 Department of Chemistry, University College in Al-Qunfudah, Umm Al-Qura University, Makkah Al-Mukarramah 1109, Saudi Arabia; 14 Botany and Microbiology Department, Faculty of Science (Boys), Al-Azhar University, Nasr City 11884, Cairo, Egypt; 15 Pathology Department, Faculty of Medicine, Zagazig University, Zagazig 44519, Al-Sharqiya, Egypt; 16 Pharmacology Department, Faculty of Medicine (for Girls), Al-Azhar University, Nasr City 11754, Cairo, Egypt; 17 Biochemistry Department, Faculty of Pharmacy, Ain Shams University, Abassia 11566, Cairo, Egypt

**Keywords:** silver nanoparticles (AgNPs), platelet-rich plasma (PRP), lung/pulmonary fibrosis (PF), *TWIST1*, *caspase* 3, CD68, iNOS (NOS2), in silico/bioinformatics analysis

## Abstract

Platelet-rich plasma (PRP) has become an increasingly valuable biologic approach for personalized regenerative medicine because of its potent anti-inflammatory/healing effects. It is thought to be an excellent source of growth factors that can promote tissue healing and lessen fibrosis. Although this treatment has demonstrated effectiveness in numerous disease areas, its impact on pulmonary fibrosis (PF) caused by silver nanoparticles (AgNPs) via its antiapoptotic effects remains to be explored. AgNPs were synthesized biologically by *Bacillus megaterium* ATCC 55000. AgNP characterization was carried out via UV–Vis spectroscopy, X-ray diffraction (XRD), dynamic light scattering (DLS), transmission electron microscopy (TEM), and scanning electron microscopy (SEM) imaging to reveal monodispersed spheres with a mean diameter of 45.17 nm. A total of 48 male Wistar rats divided into six groups, with 8 rats per group, were used in the current study on the basis of sample size and power. The groups used were the PRP donor, control, AgNP, AgNP + PRP, AgNP + dexamethasone (Dexa) rat groups, and a recovery group. Body weights, hydroxyproline (HP) levels, and *CASP3* and *TWIST1* gene expression levels were assessed. H&E and Sirius Red staining were performed. Immunohistochemical studies for inducible nitric oxide synthase (iNOS) and cluster of differentiation 68 (CD68) with histomorphometry were conducted. A significant reduction in body weight (BWt) was noted in the AgNP group compared with the AgNP + PRP group (*p* < 0.001). HP, *CASP3*, and *TWIST1* expression levels were significantly increased by AgNPs but decreased upon PRP (*p* < 0.001) treatment. Compared with those in the control group, the adverse effects of AgNPs included PF, lung alveolar collapse, thickening of the interalveolar septa, widespread lymphocytic infiltration, increased alveolar macrophage CD68 expression, and iNOS positivity in the cells lining the alveoli. This work revealed that PRP treatment markedly improved the histopathological and immunohistochemical findings observed in the AgNP group in a manner comparable to that of the Dexa. In conclusion, these results demonstrated the therapeutic potential of PRP in a PF rat model induced via AgNPs. This study revealed that PRP treatment significantly improved the histopathological and immunohistochemical alterations observed in the AgNP-induced group, with effects comparable to those of the Dexa. In conclusion, these findings highlight the therapeutic potential of PRP in a rat model of AgNP-induced PF.

## 1. Introduction

### 1.1. Background

Pulmonary fibrosis (PF) is an incurable and progressive condition leading to lung adenocarcinoma or squamous cell carcinoma [1]. PF affects the flexibility of the alveoli and is characterized by infiltration of inflammatory cytokines and apoptosis, which might result in tissue hypoxia [2] and increased damage. The first-line treatment for PF is oral administration of glucocorticoids (dexamethasone (Dexa)), which are anti-inflammatory and antifibrotic [2]. Glucocorticoids cause unfavorable adverse reactions and impede the disease’s clinical progression; therefore, safe alternative treatments are a top priority. Moreover, if these alternative treatment options are natural, they would be better.

The “personalized regenerative medicine (PRM)” umbrella, rather than the one-size-fits-all treatment [3,4], involves the use of platelet-rich plasma (PRP). PRP has potential therapeutic effects through altering cellular activities [5], increasing cell proliferation, and enhancing cellular reconstruction in chondrocytes [6], fibroblasts [7], endometrial cells [8], and corneal cells [9]. PRP is currently a favorable, popular treatment option for many medical conditions because of its biosafety and autologous nature as well as its ease of collection and application [10].

Recently, nanotechnology has been used in a wide range of life sectors, such as medicine, diagnosis, pharmaceuticals, and beauty products [11,12]. Silver (Ag) nanoparticles (NPs) are widely used in electronics, food packaging, fabrics, water purification systems, and biomedical applications [13,14]. However, AgNPs interact with biological targets because of their small size (1–100 nm), making them easier than drugs of comparable sizes. This enables them to cross the normal plasma membrane barrier [15], contributing to the use of AgNPs for wound care, catheters, and dental supplies [16], as well as other uses, but, unfortunately, would contribute to their potential toxic effects [17,18].

### 1.2. Problem Statement/Hypothesis

One of the crucial organs exposed to NPs is the lung [19]. Insoluble NPs contribute to tissue damage, inflammation, and lung tumors, according to different reports [20,21]. To avoid the adverse effects of glucocorticoid use for PF treatment or AgNP-induced lung fibrosis injury, it is crucial to identify an alternative effective, safe, and more environmentally friendly, personalized treatment [22].

### 1.3. Aim

We examined whether PRP had better or comparable antifibrotic/antiapoptotic and regenerative abilities in the AgNP-induced PF experimental model than Dexa did. Furthermore, whether PRP mitigates AgNP-induced PF via the regulation of the fibrosis/apoptosis axis was experimentally investigated via bioinformatics analysis. The findings of the present study could lead to an innovative, personalized, relatively safe treatment option for PF.

## 2. Results

### 2.1. AgNP Characterization Results

**Figure 1:**
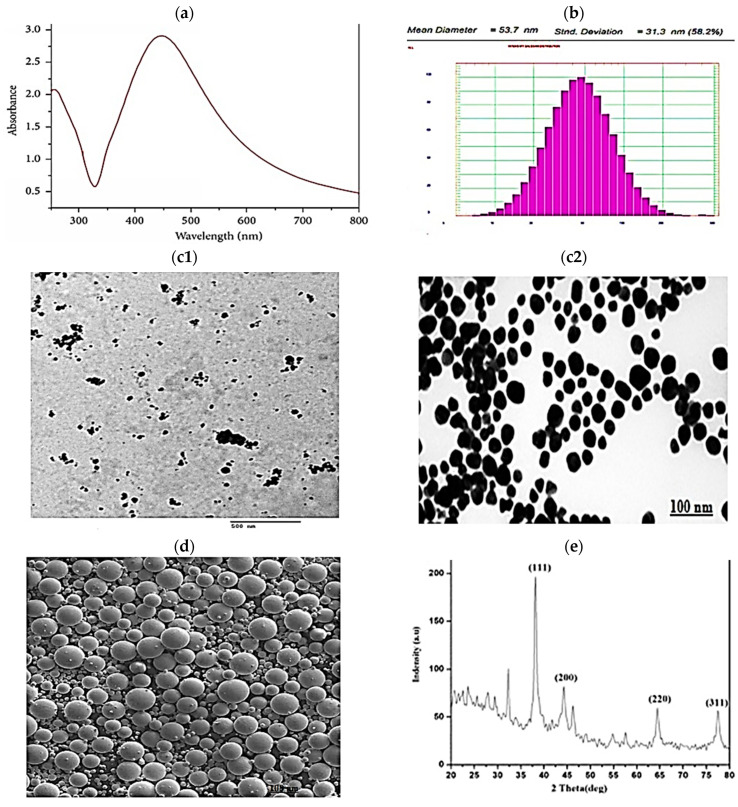
AgNP characterization via (**a**) UV–visible spectroscopy, (**b**) DLS, and (**c1**,**c2**) transmission electron microscopy (TEM) of (**c1**,**c2**), with scales of 500 and 100, respectively, (**d**) scanning electron microscopy (SEM) and (**e**) X-ray diffraction (XRD).

As explained in Figure 1a, the UV–visible spectrum of the synthesized AgNPs presented a maximum absorbance (1.312 abs) at 401.5 nm. The average particle size, as determined by dynamic light scattering (DLS), is 53.7 nm, as illustrated in Figure 1b.

The transmission electron microscopy (TEM) results demonstrated that the size of the AgNPs ranged from 26.47 nm to 63.25 nm, with an average main diameter of 45.17 nm, as shown in Figure 1(c1,c2).

By scanning electron microscopy (SEM) imaging, the AgNPs were observed to have a spherical shape, all within the nanoscale range (Figure 1d).

The diffraction pattern produced by X-ray diffraction (XRD) provided information about the crystalline nature of the synthesized AgNPs (Figure 1e). The diffraction peaked at 2θ values of 38.1°, 44.3°, 64.4°, and 77.4°, corresponding to the 111, 200, 220, and 311 planes, respectively, of the face-centered cubic (fcc) structure of silver (JCPDS No. 04-0783). It is noteworthy to mention that no peaks related to impurities were observed.

### 2.2. Bioinformatics/In Silico Analysis (Figure 2)

#### 2.2.1. DEGs from Online Datasets in Lung Cancer

**Figure 2:**
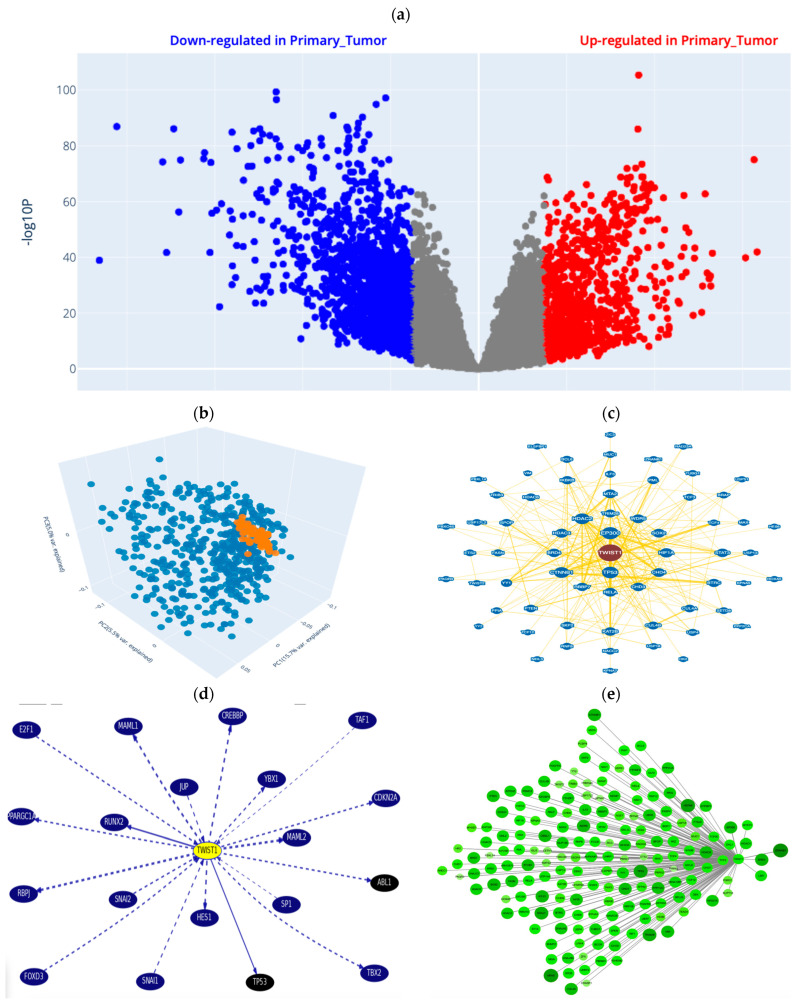

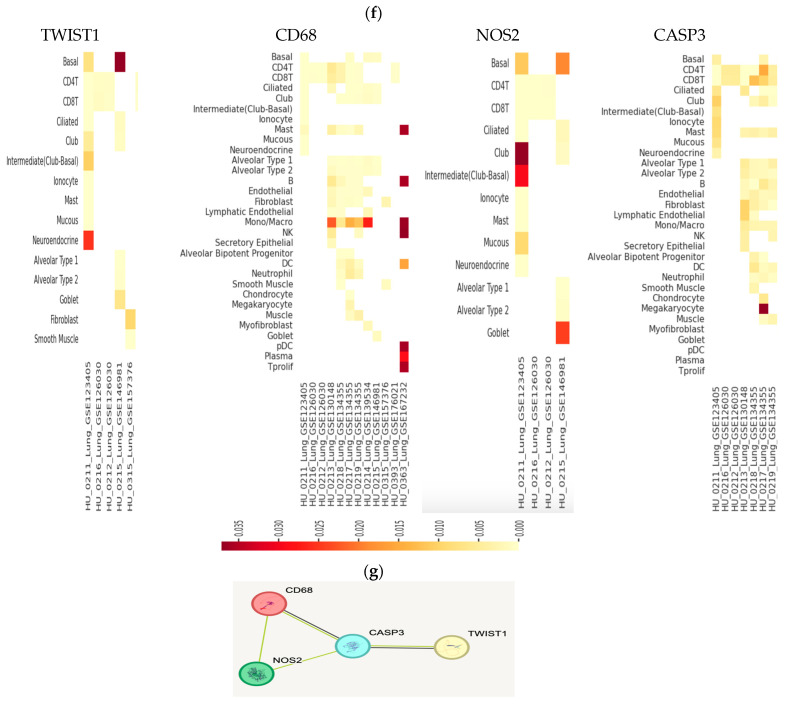
In silico search and bioinformatics analysis; (**a**) volcano plot for DEGs where upregulated genes in lung carcinoma of primary tumor vs. solid tissue normal, info (log transformed data, base 2 exponentiation is applied, all upregulated genes, are presented in red color and downregulated genes are presented in blue color), (**b**) 3D plot for PCA (a set of linearly uncorrelated variables that capture the most significant variance in the dataset is visualized via a scatter plot to illustrate sample relationships), (**c**) BioGRID^4.4^, (**d**) (http://genome.ucsc.edu/cgi-bin/hgGeneGraph?gene=TWIST1&1=OK&supportLevel=pwy&hideIndirect=on&geneCount=20&hgsid=2545836050_aoRiUiKKxAWYBfX8MdRsxB6DAq1A&geneAnnot=drugbank&geneCount=15&1=OK, accessed on 8 May 2025) and (**e**) ctd gene–gene interaction. (https://ctdbase.org/tools/cyjs.go?type=gene&report=gg&window=yes&color=91&terms=7291, accessed on 8 May 2025) (**f**) *TWIST1*, *CD68*, *NOS2*, and *CASP3* relative expression heatmap within the lung tissue/cells from the Human Universal Single-Cell Hub (http://husch.comp-genomics.org/#/info_tissue/Lung, accessed on 8 May 2025) with the highly expressed genes in the dark red color and the lowest expression in the pale yellow color, (**g**) through the STRING 12.0 database; the link between CD68 and NOS2 with *TWIST1* through the *CASP3* link (https://string-db.org/, accessed on 8 May 2025).

To retrieve relevant gene expression data related to PF, we accessed the UCSC Xena Browser (https://xenabrowser.net, accessed on 8 May 2025) to select the dataset (TCGA lung adenocarcinoma or lung squamous cell carcinoma). The first variable was phenotypic sample types, such as primary tissue vs. normal solid tumors (https://xenabrowser.net/heatmap/, accessed on 8 May 2025) to compare the expression of different genes in available online datasets via the Xena Differential Gene Expression Analysis Pipeline (https://github.com/ucscXena, accessed on 8 May 2025) to perform Differential Gene Expression (DGE) analysis and further downstream analyses. The second variable was antiapoptotic gene expression (Figure 2a). Converting gene expression values into a principal component 3D plot for PCA is shown in Figure 2b. Moreover, the genes with their Log2FC, average expression, and *p* value are attached as a Supplementary Excel File.

#### 2.2.2. Data Sourcing 

TWIST1 is a protein-coding gene located on chromosome 7; 7p21.1 (−1 strand) and via the Clinical Interpretations of Variants in Cancer (CiViC) v2.2.33 (https://civicdb.org/features/5964/summary, 13 December 2023). TWIST1 inhibits the hypoxia-inducible factor-2-alpha (HIF-2-alpha) transcription factor network, cytokine signaling in the immune system, disease, immune system, generic transcription pathway, signaling by interleukins, disease signal transduction by growth factor receptors and second messengers, interleukin-4 and interleukin-13 signaling, RNA polymerase II transcription, gene expression (transcription), regulation of RUNX2 expression and activity, and more biological processes/pathways from biocarta, KEGG, pid, gene ontology (GO) (https://platform.opentargets.org/target/ENSG00000122691, accessed on 8 May 2025). and reactome sources.

#### 2.2.3. Data Processing Results

##### Gene–Gene and Protein–Protein Interactions (PPIs)

*TWIST1* is expressed in and related to myeloid dendritic cells and memory T-regs, where it has antiapoptotic effects. TWIST1 interacts with the tumor suppressor protein transcription factor p53 (which is inhibited by acetylsalicylic acid) and regulates p53 target gene expression (Figure 2c) via BioGRID^4.4,^ as confirmed via UCSC (http://genome.ucsc.edu/cgi-bin/hgGeneGraph?gene=TWIST1&1=OK&supportLevel=pwy&hideIndirect=on&geneCount=20&hgsid=2545836050_aoRiUiKKxAWYBfX8MdRsxB6DAq1A&geneAnnot=drugbank&geneCount=15&1=OK, accessed on 8 May 2025) (Figure 2d) for gene interactions and pathways from curated databases and text mining. This finding was confirmed via the Comparative Toxicogenomics Database (ctd) for gene–gene interactions shown in Figure 2e, where the ctd database integrates gene–gene and protein–protein interactions from BioGRID (https://ctdbase.org/detail.go?type=gene&acc=7291, accessed on 8 May 2025). 

Relative expression heatmaps of the *TWIST1, CD68, NOS2*, and *CASP3* genes within the lung tissue/cells from the Human Universal Single-cell Hub (http://husch.comp-genomics.org/#/info_tissue/Lung, accessed on 8 May 2025) (Figure 2f).

Finally, as shown in Figure 2g, via STRING v12.0, the link between NOS2, CD68, and TWIST1 through CASP3 was confirmed.

### 2.3. Changes in Body Weight (BWt) in the Different Groups

**Figure 3:**
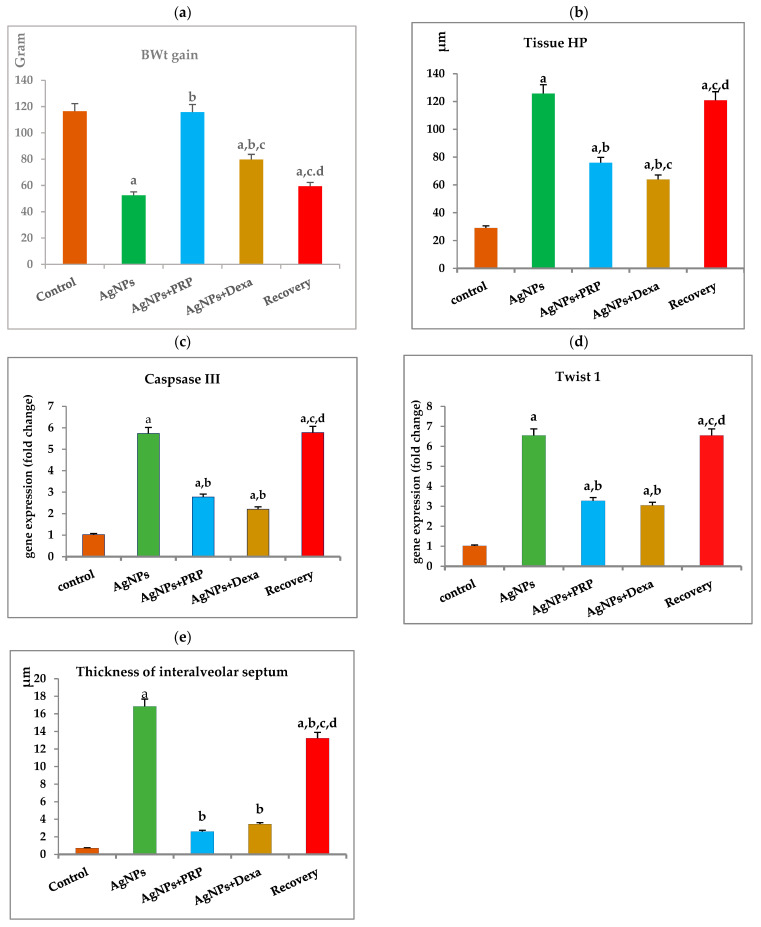
(**a**) Body wt gain in the different groups, (**b**) lung tissue hydroxyproline (HP) in the different groups, (**c**) lung relative expression levels of the *CASP3* gene in the different groups, (**d**) lung relative expression levels of the *TWIST1* gene in the different groups, and (**e**) thickness of the interalveolar septum (µm) in the different groups. ANOVA and the Bonferroni post hoc correction were employed to identify significant differences via SPSS for Windows (v23.0; IBM Corporation, Armonk, NY, USA). The results are presented as the means ± SDs. Groups with different superscripts are significantly different (*p* < 0.05). [a: control group; b: Ag-NP group; c: Ag-NP + PRP group; d: Ag-NP + Dexa group] (n: number/group).

Figure 3a shows that a lower BWt gain was observed in the AgNP group than in the control group (*p* < 0.001). A greater BWt gain was detected in the AgNP + PRP group than in the AgNP group (*p* < 0.001).

Notably, no mortality, gross effects, or significant differences in nourishment were observed during the study period in any of the rats that were administered AgNPs compared with those in the control group. However, treated rats presented a marked decrease in activity.

### 2.4. Levels of the Lung Tissue Fibrosis Marker Hydroxyproline (HP) in the Different Groups

As depicted in Figure 3b, the HP level was greater (*p* < 0.001) in the AgNP group than in the control group by approximately 4.3-fold. A lower HP level of approximately 49% was observed in the AgNP + Dexa group than in the AgNP group. A substantial (*p* < 0.001) decrease in the HP level of approximately 40% was also observed in the AgNP + PRP group compared with the AgNP group.

### 2.5. PRP Alleviates the Expression of the Apoptotic Marker CASP3 and the Fibrosis Indicator TWIST1

As shown in Figure 3c, the level of CASP3 was 5.6-fold greater in the AgNP group than in the control group. Moreover, significant downregulation (*p* < 0.001) was observed in the AgNP + PRP and AgNP + Dexa groups, with reductions of approximately 52% and 61%, respectively, compared with those in the AgNP group. A similar trend was noted for TWIST1 (Figure 3d), which was significantly elevated by approximately 6.5-fold in the AgNP group compared with the control group. In contrast, the TWIST1 level was significantly lower in the AgNP + PRP and AgNP + Dexa groups than in the AgNP group by approximately 60.5% and 53.6%, respectively.

These findings suggest that PRP has antiapoptotic and antifibrotic potential against AgNP-induced PF.

### 2.6. Histological Studies Results

#### 2.6.1. Hematoxylin and Eosin (H&E) Staining

**Figure 4:**
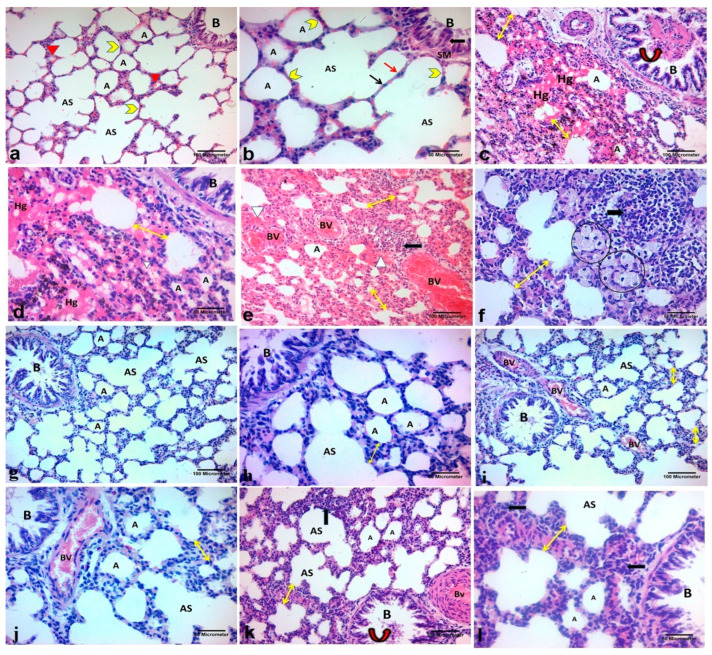
Photomicrographs of H&E-stained lung sections from the different study groups. (**a**,**b**) Control group: normal lung architecture, formed of normal alveoli (A), alveolar sacs (AS), thin interalveolar septa (bifid yellow arrowhead), bronchioles (B), and pulmonary blood vessels (red arrowheads). (**b**): Alveae are lined by squamous type I pneumocytes with flattened nuclei (thin black arrow) and cuboidal type II pneumocytes with rounded nuclei (red arrow). The bronchioles (B) are lined with simple columnar epithelium (thick black arrow) and surrounded by a smooth muscle layer (SM). (**c**,**d**): AgNP group: narrowed alveolar spaces (A), thick interalveolar septa (double head arrow), multiple, large areas of hemorrhage (Hg), extravasated RBCs (white arrowheads) within the lung interstitium, and detached cells with darkly stained nuclei (curved arrows) in the bronchiolar lumen; (**e**): thick interalveolar septa (double head arrow), congested dilated blood vessels (BVs) and lymphocytes. infiltration (black arrow); (**f**): thick interalveolar septa (double head arrow), large alveolar macrophages (circles) with vacuolated acidophilic cytoplasm in the pulmonary interstitium, and massive masses. lymphocytic. (black arrow). (**g**,**h**) In the AgNP + PRP group, the histological structure of the lung was similar to that of the control group, with normal alveoli (A), alveolar sacs (AS), and thin interalveolar septa and pulmonary bronchioles (B). However, thick interalveolar septa (double head arrow) in some regions can be observed in the (**i**,**j**) AgNP + Dexa group, potentially alleviating lung architecture and thickening of the interalveolar septa (double head arrow) in some regions with congested dilated blood vessels (BV). (**k**,**l**) Recovery group: narrowed alveolar spaces (A), thick interalveolar septa (double head arrow), detached cells with darkly stained nuclei (curved arrow) in the bronchiolar lumen (B), and congested pulmonary blood vessels (BV) with some lymphocytic infiltration (black arrows). (H&E (**a**,**c**,**e**,**g**,**i**,**k**) ×200; scale bar = 100 μm; (**b**,**d**,**f**,**h**,**j**,**l**) ×400; scale bar = 50 μm).

The results of the H&E staining used to examine the lung tissue in the different groups are shown in Figure 4a–l. Lung sections from the AgNP and recovery groups revealed a loss of the normal architecture of the lung tissue in the form of numerous and enormous hemorrhagic areas, destroyed and narrowed alveoli with noticeable thickening of the interalveolar septa, and extravasated red blood cells (RBCs) within the lung interstitium. Large alveolar macrophages with vacuolated acidophilic cytoplasm, congested dilated pulmonary blood vessels, extensive lymphocytic infiltration, and epithelial desquamation were observed in a few bronchioles. Compared with the AgNP group, the AgNP + PRP group presented significant structural improvement, and the typical lung architecture of the control group was restored.

Dexa therapy in the AgNP + Dexa group appeared to decrease the thickness of the interalveolar septa. Compared with those in the AgNP group, there was also less cellular infiltration and a wider alveolar lumen. However, several congested blood vessels and interalveolar septal thickening still occur in some regions.

#### 2.6.2. Sirius Red Stain

**Figure 5:**
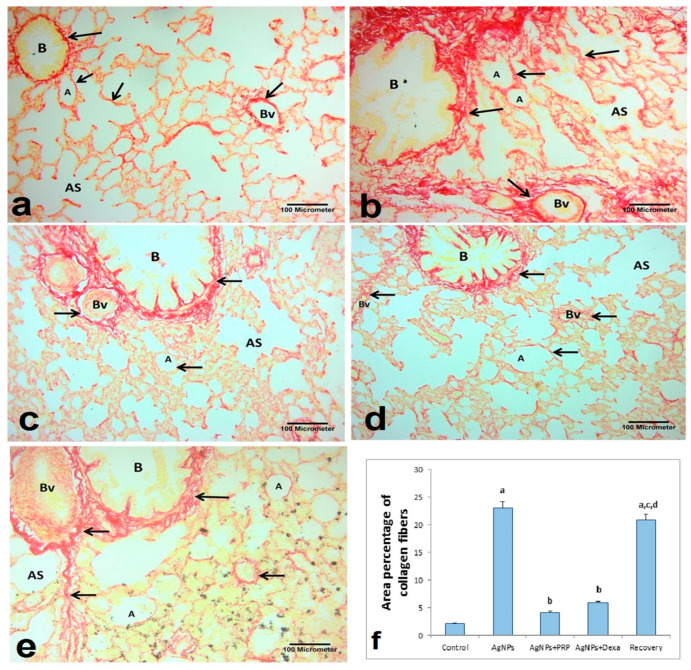
Photomicrographs of Sirius Red-stained lung sections from different groups. (**a**) Control group: minimal collagen fibers within the lung interstitium (black arrows), between alveoli (A), around the alveolar sac (AS), in the thin interalveolar septum, around the blood vessel wall (Bv), and around the pulmonary bronchioles. (**b**) AgNP group: excessive collagen fibers deposited around the walls of the blood vessels (Bv), around the pulmonary bronchioles (B), between the alveoli (A), and around the alveolar sac (AS). (**c**) AgNP + PRP group: minimal collagen fibers within the lung interstitium (black arrows), between alveoli (A), around the alveolar sac (AS), in the thin interalveolar septum, around the blood vessel wall (Bv), and around pulmonary bronchioles (B), as in the control group. (**d**) AgNP + Dexa group: decreased deposition of collagen fibers around the walls of blood vessels, around pulmonary bronchioles (B), between alveoli (A) and around the alveolar sac (AS). (**e**) Recovery group: excessive collagen fibers were deposited around the wall of the blood vessel (Bv), around the pulmonary bronchioles (B), between the alveoli (A), and around the alveolar sac (AS). (**f**): Quantification of the percentage of collagen fibers in the lung tissue. The mean percentage area of collagen fibers was measured in ten randomly selected microscopic fields and averaged for each animal. The bars represent the means ± SDs. Groups with different superscripts are significantly different (*p* < 0.05). [a: control group; b: Ag-NP group; c: Ag-NP + PRP group; d: Ag-NP + Dexa group]. (Sirius Red stain; ×200, scale bar = 100 μm).

Small strands of collagen fibers could be observed in the thin interalveolar septum surrounding the blood vessels, around the pulmonary bronchioles, and around the alveoli in Sirius Red-stained slices from the control group. The collagen fiber distribution and density clearly increased in some of the AgNP-treated and recovery groups. The thickened interalveolar septa, the area around the bronchioles, and the area around the clotted blood all showed signs of interstitial fibrosis, as shown in Figure 5. In contrast, lung tissue sections from the AgNP + PRP and AgNP + Dexa groups presented reduced collagen fiber deposition around blood vessels, pulmonary bronchioles, alveoli, and alveolar sacs, as well as thin interalveolar septa. This reduction was more pronounced in the AgNP + PRP group, which closely resembled that of the control group. These findings align with the results of the biochemical analysis, indicating the downregulation of the TWIST1 gene and the lower HP content in lung tissues.

### 2.7. Immunohistochemistry Results

#### 2.7.1. iNOS

**Figure 6:**
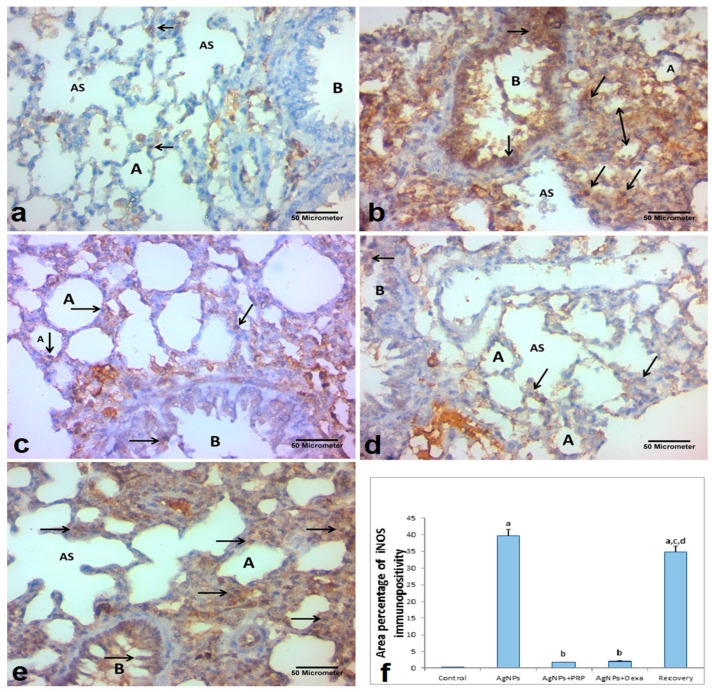
IHC staining of iNOS in lung sections from the experimental groups (x400); iNOS appeared as brown cytoplasmic immunoreactions. (**a**) Control group: a few immunoexpressions of iNOS (arrows) within the alveolar wall (A), alveolar sac (AS) cells, and bronchiolar epithelial lining (B). (**b**) AgNP group: strong positive immunoexpression of iNOS (arrows) within the alveolar wall (A), alveolar sac (AS), bronchiolar epithelial lining (B), and thickened interalveolar septum (double head arrow). (**c**) AgNP + PRP group: few immunoexpressions of iNOS (arrows) within the alveolar wall (A) and bronchial epithelial lining, more or less than those of the control group. (**d**) AgNP + Dexa group: decreases in the expression of iNOS (arrows) within most cells of the alveolar wall (A), alveolar sac (AS), and bronchial epithelial lining (B). (**e**) Recovery group: showing marked immunoexpression of iNOS (arrows) within the cells of the alveolar wall (A), alveolar sac (AS), and bronchial epithelial lining (B). (**f**): Quantification of iNOS expression in lung tissue. The mean percentage area of iNOS immunopositivity was measured in ten randomly selected microscopic fields and averaged for each animal. The bars represent the means ± SDs. Groups with different superscripts are significantly different (*p* < 0.05). [a: control group; b: Ag-NP group; c: Ag-NP + PRP group; d: Ag-NP + Dexa group].

Figure 6 shows the presence of a few cytoplasmic immunoreactions of iNOS in the bronchioles and lung interstitial cells in the control group. Compared with the control group, both the AgNP-treated group and the recovery group presented strong positive dark brown iNOS immunoreactions that revealed apoptotic alterations in the lung tissue and were evidently apparent in the cytoplasm of the alveolar epithelial cells, interalveolar septa, interstitial cell populations, and bronchiolar epithelial lining.

Like the control group, the AgNP + PRP group presented a few and sparse cytoplasmic immunoreactions of iNOS among the cells of the lung interstitium and bronchioles. There was a noticeable decrease in iNOS expression in the AgNP + Dexa group. A weak positive brown cytoplasmic immunoreaction for iNOS was found in the pulmonary interstitial cells, cells of the alveolar epithelium, and the bronchiolar epithelial lining in the AgNP + PRP and AgNP + Dexa groups.

#### 2.7.2. Alveolar Macrophage IHC Response to Cluster of Differentiation 68 (CD68)

**Figure 7:**
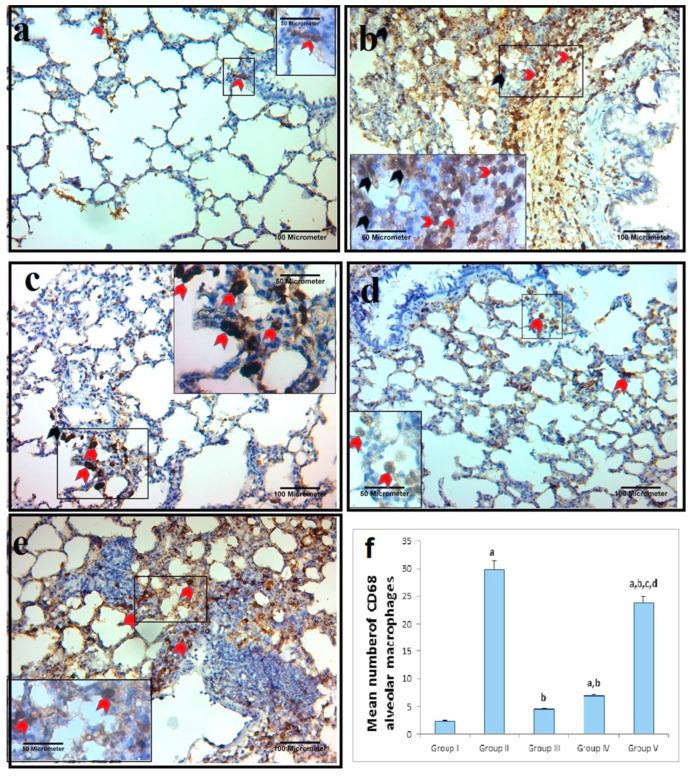
IHC staining of CD68 in lung sections from different groups. (**a**) Control group: a few brown positive-stained CD68-positive alveoli (red arrowhead). (**b**) AgNP group: There were marked dark brown positively stained CD68+ alveolar macrophages within the interalveolar septa (black arrowhead) and in the wall of the bronchi (red arrowhead). (**c**) AgNP + PRP group: **a** few brown positive-stained CD68+ alveolar macrophages (red arrowhead) were observed in the control group. (**d**) AgNP + Dexa group: decrease in brown-positive CD68+ alveolar macrophages (red arrowhead). (**e**) Recovery group: there were marked dark brown positively stained CD68+ alveolar macrophages within the interalveolar septa (red arrowhead). (**f**) Quantification of CD immunoexpression in lung tissue. The number of CD immunopositivity events was measured in ten randomly selected microscopic fields and averaged for each animal. The bars represent the means ± SDs. Groups with different superscripts are significantly different (*p* < 0.05). [a: control group; b: Ag-NP group; c: Ag-NP + PRP group; d: Ag-NP + Dexa group]. (CD68 immunohistochemical staining ×200; scale bar = 100 μm. With inset ×400; scale bar = 50 μm).

Only a small percentage of the interalveolar macrophages within the control group exhibited a slightly positive cytoplasmic immunoreaction to CD68, as depicted in Figure 7. However, compared with those in the control group, the interalveolar septa and bronchial wall of the AgNP-treated and recovery groups presented a significant number of cells with positive cytoplasmic immunoreaction. The AgNP + PRP group presented a considerable decrease in positively stained CD68 immunoreactivity, while it was still similar to that of the control group. Compared with the AgNP + Dexa group, the AgNP + Dexa group presented a discernible decrease in the number of strongly stained CD68 immunoreactions. Immunoreactions manifested as mildly positive cytoplasmic immunoreactions in cells of the interalveolar septa.

#### 2.7.3. Morphometric Analysis

Figure 3e shows that the AgNP group and the recovery group had significantly thicker interalveolar septa, on average, by approximately 23- and 18-fold, respectively, than did the control group under H&E staining (*p* ˂ 0.001). In terms of morphometric aspects, the AgNP + PRP group and the AgNP + Dexa group showed substantial improvements compared with the AgNP group.

## 3. Discussion

Currently, there are a variety of effective treatments for PF, including pirfenidone, N-acetylcysteine, and prednisone, which suppress collagenase activity and alveolar type II pneumocyte proliferation [23], increasing the risk of developing pneumonia or pulmonary tuberculosis [24] and lung cancer [25]. Therefore, finding safe alternative treatments for PF or lung injury from interactions with environmental NP exposure [25] is necessary.

BWt is frequently employed as a sensitive marker of chemical toxicity [26]. Our results were consistent with those of El-Naggar et al. (2021) [27], Tiwari et al. (2011) [28], Yin et al. (2015) [29], and Xia et al. (2014) [30], who demonstrated that administering AgNPs at concentrations of 20 and 40 mg/kg decreased the BWt. However, no apparent changes in BWt were reported by Ma et al. (2020) [31] when rats were exposed to AgNPs (200 μL, 1 mg/mL), through a single intratracheal instillation. On the other hand, the BWt of the rats increased upon exposure to AgNPs at doses of 100, 1000, and 5000 mg/kg per day for seven, fourteen, and twenty-one days, respectively, in one study [32].

Compared with the AgNP and AgNP + Dexa groups, the PRP (0.5 mL kg^−1^ BWt i.p.) group presented significantly greater BWt values. These results support the findings of Xu et al. (2018) [33] and Tong et al. (2018) [34], who demonstrated that PRP reduced weight loss in diabetic rats. The effectiveness of this therapy lies in its content of growth factors and proteins that mimic and assist physiological tissue reconstruction [35] through cell cycle progression by promoting cyclin expression [36] and its proliferative effect on preadipocytes [37].

HP was used as a PF marker to assess the effect of PRP on AgNP-induced PF [27,38]. Compared with those in the control group, the HP levels in the AgNP-treated group significantly increased. An improvement in lung tissue architecture and a dramatic reduction in the distribution of collagen fibers were revealed by histological assessment of lung tissue sections from PRP-treated rats. Dexa treatment reduced HP levels more than PRP did; however, both treatments showed comparable efficacies, which is in line with the findings of Salem et al. (2018) [39,40]. PRP supports the body’s innate healing mechanisms by delivering platelets and various CDs to the injured location and attracting stem cells as an initial response to the injury [41]. These proteins are known to maintain membrane integrity, promote endothelial development, decrease membrane permeability, activate intracellular signaling pathways, and trigger regenerative process transcription [42,43,44]. Notably, owing to their small size, AgNPs are able to infiltrate the intracellular environment, where they can prevent cellular development and trigger apoptosis and necrosis [45,46,47,48].

The degree of lung damage caused by subchronic exposure to AgNPs was also assessed at the molecular level by analyzing the activation of the *CASP3* and *TWIST1* genes. The current study demonstrated that following AgNP administration, the *CASP3* and *TWIST1* genes were significantly upregulated compared with those in the control group. PRP injection caused significant downregulation of the *CASP3* and *TWIST1* genes, similar to Dexa therapy. Our findings were consistent with those of Sekerci et al. (2017), who reported that PRP lowered *CASP3* levels in rats [49].

The histological analysis of lung tissues in this work confirmed that PRP had a reasonable antiapoptotic effect. *CASP3* overexpression is interpreted as an increase in apoptosis [50]. Exposure to NPs reduces mitochondrial activity, which leads to an increase in apoptosis [51]. The release of cytochrome c from the mitochondria activates *CASP3*, which causes the creation of an apoptosome [52]. Caspases such as *CASP3* may be activated by oxidative stress, which enhances cell death [53]. AgNPs cause apoptosis via the caspase-dependent mitochondrial pathway, altering cell dynamics, damaging the cell membrane, inactivating ATPase, producing excessive reactive oxygen species, and activating the caspase cascade [54,55].

Hunyenyiwa et al. (2021) reported that *TWIST1* and other E-box transcription factor motifs were significantly upregulated in the PF of bleomycin-injured mice [56]. This upregulation was also associated with increased collagen synthesis, which aligns with our results. *TWIST1* is related to *TP53*, hypoxia inducible factor, and other transcription factors that are related to apoptosis, autophagy, and death, as revealed by our bioinformatics results.

Moreover, the current work revealed that AgNP-treated lung sections presented increased iNOS expression due to the production of proinflammatory factors that catalyze arginine breakdown and nitric oxide production, leading to severe oxidant (ONOO) damage and DNA and cytomembrane oxidation [57,58].

Compared with that of the control group, the average quantity of CD68+ immunoreactive cells in the lung sections of the AgNP group was notably greater. This could be explained as a compensatory mechanism to protect normal lung function from the risk of the inflammatory cascade [59]. Compared with that in the AgNP-treated group, a significant improvement in the histological architecture of the lung tissues was observed in the AgNP + PRP group. This occurred because the growth factors in PRP and platelet stimulation promote stem cell proliferation, leading to lung tissue regeneration [60], an enhanced natural healing process [61], and increased cyclic adenosine monophosphate (cAMP) levels, which are known for their antifibrotic effects [62].

The molecular, histological, and IHC results were confirmed by the STING database results connecting CD68 and NOS2 to *TWIST1* through *CASP3*.

## 4. Materials and Methods

### 4.1. Drugs, Chemicals, Reagents, Antibodies, and Kits

Dexamethasone injectable vials containing 8 mg/2 mL (Pharm. Inco. Amryia, Cairo, Egypt). *Bacillus megaterium* ATCC 55000 culture, PVDF filter, silver nitrate solution, deionized water, sodium citrate, phosphate-buffered saline (PBS), calcium chloride (CaCl_2_), and sodium pentobarbital were used. The other substances or reagents/solvents were of the highest quality. Rat HP ELISA kits (Hangzhou East Biopharma Co., Ltd., Hangzhou, China), an RNA Qiagen tissue extraction kit (Qiagen, Hilden, Germany), a high-capacity cDNA reverse transcription kit (Fermentas, Thermo Fisher Scientific, Waltham, MA, USA), SYBR Green Master Mix (Applied Biosystems, Foster City, CA, USA), an iNOS-recognizing primary antibody (rat monoclonal antibody, 1:500 dilution; Transduction Laboratories, San Diego, CA, USA), and a CD68-recognizing primary antibody (mouse monoclonal antibody, 1:200 dilution; NCL-L-CD68; Leica Biosystems, Benton La, Newcastle Ltd., Balliol Business Park, Benton Ln, Newcastle upon Tyne, UK) were used. 

### 4.2. AgNP Biosynthesis

*Bacillus megaterium* ATCC 55000 cultures were subcultured on nutrient broth media in conical flasks and incubated with shaking aerobically at 37 °C for 48 h. After the incubation period, the bacterial cells were removed from the suspension by filtration through a 0.44 _m PVDF filter; then, they were centrifuged at 10,000 rpm to remove occasional bacterial cells and macromolecules. An aqueous solution of 1 mM silver nitrate solution (50 mL) was mixed with bacterial supernatants (50 mL), and the pH was adjusted to 8.5. The mixture was incubated in a rotary shaker at 37 °C and 200 rpm in the dark for 24 h. Control experiments were performed with uninoculated media and silver nitrate solution to determine the role of bacteria in NP synthesis. Silver ion reduction was examined by sampling approximately 2 mL of the solution at time intervals and monitoring the UV–Vis spectra via a UV–Vis spectrophotometer (JASCO V-560) (Tokyo, Japan). During each reaction, a change in vessel color was observed, resulting in the formation of a white suspension. The AgNPs were further centrifuged at 12,000 rpm for 30 min, and the collected precipitate pellet was dried and weighed.

### 4.3. The AgNP Suspension Was Purified via Centrifugation [63,64]

Centrifugation: The synthesized AgNPs were pelleted by subjecting the suspension to high-speed centrifugation at 12,000 rpm for 30 min. This step concentrates the nanoparticles by separating them from the reaction medium.

Supernatant removal: The supernatant, which contains unreacted precursors, soluble impurities, and any residual biological materials (e.g., proteins or enzymes), was carefully discarded to minimize contamination.

Pellet resuspension: The resulting nanoparticle pellet was resuspended in distilled water.

Washing steps: To ensure thorough purification, the centrifugation and resuspension steps were repeated 2–3 times. This iterative washing helps remove residual contaminants and stabilizes the AgNPs in the desired medium. Finally, the collected precipitate pellet was dried and weighed.

### 4.4. AgNP Characterization 

Characterization of the AgNPs was performed by using a JASCO V-560 UV–Vis spectrophotometer (Tokyo, Japan) at wavelengths ranging from 200 to 900 nm and at a resolution of 1 nm with cell-free supernatant without the addition of zinc nitrate as a baseline blank (negative) for autozero support. For the particle size investigation, the samples were diluted tenfold (10×) prior to analysis with deionized water before estimation. The morphology and size of the manufactured AgNPs were determined via DLS, TEM, and scanning electron microscopy SEM (JEOL JEM-100 CX, Peabody, MA, USA).

TEM imaging was scouted by a drop covering the AgNPs on the carbon-coated TEM layers. The samples were subsequently centrifuged before XRD examination, after which they were transferred to the recovered precipitate, after which they were subsequently centrifuged and drained below vacuity. XRD was performed with a Shimadzu instrument (XRD-6000 line; Tokyo, Japan). The estimations included stress investigations, remaining austenite quantification, crystallite capacity, crystallinity considerations, and material examination through overlaid XRD models [65].

### 4.5. Bioinformatics/In Silico Analysis 

#### 4.5.1. DGE of the Different Genes from Online Datasets in Lung Cancer

To retrieve relevant gene expression data related to PF, we accessed the UCSC Xena Browser (https://xenabrowser.net, accessed on 8 May 2025).

#### 4.5.2. Data Sourcing

According to Online Mendelian Inheritance in Man^®^ (https://omim.org/entry/601622, accessed on 8 May 2025), according to the ontology search (OLS) ebi.ac.uk (https://www.ebi.ac.uk/ols4/ontologies/ncit/classes/http%253A%252F%252Fpurl.obolibrary.org%252Fobo%252FNCIT_C39917, accessed on 8 May 2025) and an orphaned (https://www.orpha.net/en/disease/gene/TWIST1?name=twist1&mode=gene, accessed on 8 May 2025) as well as cancer network genes (CNGs) (http://network-cancer-genes.org/query.php, accessed on 8 May 2025) DECIPHER GRCh38 genome browser (https://www.deciphergenomics.org/browser#q/TWIST1/location/7:18944313-19194313, accessed on 8 May 2025). The Clinical Interpretations of Variants in Cancer (CiViC) v2.2.33 (https://civicdb.org/features/5964/summary TWIST1 processes/pathways, accessed on 13 December 2023) from biocarta, KEGG, pid, gene ontology (GO) (https://platform.opentargets.org/target/ENSG00000122691, accessed on 8 May 2025), and reactome sources.

#### 4.5.3. Data Processing

Protein–protein interactions (PPIs). Through the STRING 12.0 database, the links among CD68, NOS2, *TWIST1*, and *CASP3* were explored (https://string-db.org/ accessed on 8 May 2025).

### 4.6. PRP Preparation

PRP was prepared via the double centrifugation tube method [66]. Three milliliters of blood were drawn from the retro-orbital plexus of the donor rat group. After the rats were given a sodium pentobarbital injection (30 mg kg^−1^), the whole blood was collected in a 0.3-millilitre 3.2% sodium citrate tube and centrifuged at 1500× *g* for 15 min at room temperature. The supernatant plasma and buffy coat were transferred to a sterile tube and subjected to a second centrifugation at 2800× *g* for 7 min to concentrate the platelets. The platelet-poor plasma (PPP) was partially removed, leaving 1 mL of PRR above the pellet. The platelet concentration was confirmed to be >1,000,000 platelets/μL via hematologic analysis.

The initial centrifugation ran for 10 min at 1600 rpm. Without damaging the buffy coat, the section directly above it was retrieved. Two portions were obtained from the second centrifugation, which was performed for ten minutes at 2000 rpm. The top portion included PPP, and the bottom portion contained the platelet button. The platelet pellet was resuspended in PBS (1:1) after the PPP was removed. A hematology analyzer (MICROS abc LC-152; Horiba Ltd. (Kyoto, Japan), with a minimum of one million cells per microliter, was used [67]. PRP was instantly activated by calcium chloride (CaCl_2_) (0.8 mL PRP + 0.2 mL 10% CaCl_2_) [39].

### 4.7. Experimental Design

#### 4.7.1. Sample Size and Study Power

For scientific validity as well as animal welfare, a pilot study was conducted. The differences in the BWt mean and standard deviation (SD) values at a power of 80% were used to minimize the number of animals used, and an alpha level of 0.05 (http://www.lasec.cuhk.edu.hk/sample-size-calculation.html, accessed on 8 May 2025) was used for 8 rats/group.

Animals: Male albino Wistar rats (weighing 160 ± 30 g) were provided by Helwan Breeding Farm, Helwan, Egypt. The animals were kept in an animal care facility under controlled temperature (25 ± 2 °C) and humidity (54 ± 5%) and on a 12 h/12 h light/dark cycle, with free access to tap water and standard pelleted animal food provided ad libitum. Where, a typical laboratory chow diet was provided in clean, well-ventilated wire-mesh cages (25 × 30 × 25 cm) with 2 rats in each cage. The animal facility is located at the Faculty of Medicine (Girls), Al-Azhar University, Cairo, Egypt.

#### 4.7.2. Experimental Protocol (Figure 8)

Predefined, well-characterized AgNPs were administered to rats at a dose of 10 mg kg^−1^ BWt via an intraperitoneal (i.p.) injection once daily for four successive weeks. The AgNP dose was selected because of its ability to produce considerable subchronic toxicity in albino rats, according to the study by Yousef et al. in 2012 [68].

Half a milliliter per kg BWt of the produced PRP was injected i.p. twice a week for three weeks, 24 h after the last AgNP dose [69].

The i.p. route was selected for administering AgNPs in this study because of its significantly greater precision and consistency than other routes of potentially hazardous exposure, ensuring subchronic toxicity [70].

**Figure 8:**
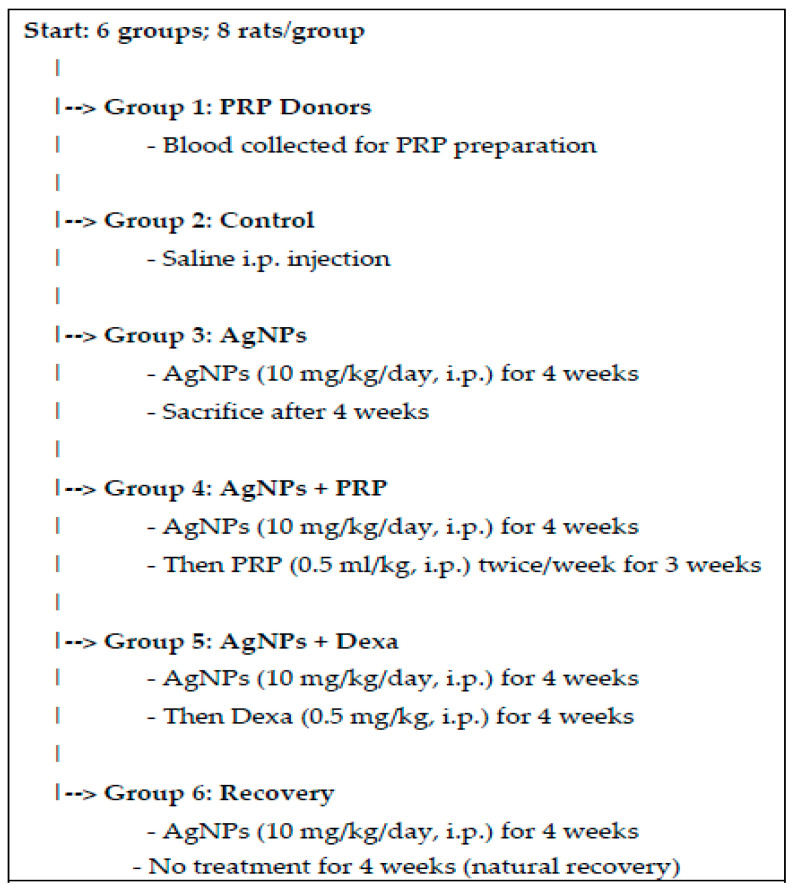
Experimental design and protocol flowchart.

The rats were randomly divided into six groups with 8 rats/group, as shown in Figure 8.

Group 1 (PRP donors) served as blood donors for the preparation of the PRP,

Group 2 (control) rats were used as controls (saline) (negative control).

Group 3 (AgNP) rats were subjected to i.p. injection of AgNPs (10 mg kg^−1^ BWt/day) for four weeks and then sacrificed [68],

Group 4 (AgNP + PRP) rats received an i.p. injection of AgNPs (10 mg kg^−1^ BWt/day) for four weeks followed by an i.p. injection of PRP at a dose of 0.5 mL kg^−1^ BWt twice weekly for three weeks, 24 h after the last AgNP dose.

Group 5 (AgNPs + Dexa) rats received AgNPs similar to those in group 3, followed by Dexa i.p. at a dose of 0.5 mg kg^−1^ BWt for four weeks, according to Chen et al., 2006 [22].

Group 6 (recovery) rats were given an i.p. injection of AgNPs (10 mg kg^−1^ BWt/day) for four weeks and left untreated for four weeks until the end of this study.

### 4.8. Body Weight

To determine BWt changes, the animals were weighed before and at the end of the experimental protocol.

### 4.9. Biochemical Assays

At the end of the experiment, the rats were euthanized via sodium pentobarbital injection (30 mg/kg), after which they were sacrificed by dislocation. The lungs were then processed for biochemical investigations of HP per lung and quantitative real-time polymerase chain reaction (qRT–PCR) to detect *CASP3* (Qiagen, Cat No. QT00088196) and *TWIST1* (Qiagen, Cat No. QT00011956) gene expression levels in the lung tissue homogenate.

#### 4.9.1. Detection of HP Levels per Lung

A rat HP ELISA kit (Hangzhou East Biopharma Co., Cat No. MBS017427 Ltd., Qiantang New Area, Hangzhou, China) was used according to the manufacturer’s instructions, and the absorbance was read at 450 nm via a plate reader.

#### 4.9.2. Detection of the Gene Expression of *CASP3* and *TWIST1* in Lung Tissue via Quantitative Real-Time Polymerase Chain Reaction (qRT–PCR)

Total RNA was isolated via a Qiagen tissue extraction kit (Qiagen, Cat No. 69504/69506, Hilden, Germany) according to the manufacturer’s instructions. Total RNA was used for complementary DNA (cDNA) synthesis via a high-capacity cDNA reverse transcription kit (Fermentas, Cat No. 4368813, Waltham, MA, USA). Moloney murine leukemia virus (MMLV) reverse transcriptase was used for the synthesis of cDNA from RNA. Human placental ribonuclease inhibitor (HPRI) was used for the inhibition of RNAase activity. Real-time qPCR amplification and analysis were performed via the Applied Biosystem with software version 3.1 (Applied Biosystems, StepOne™, Foster City, CA, USA). The reactions included SYBR Green Master Mix (Applied Biosystems, Cat No. 4472908) and gene-specific primer pairs, which are shown in Table 1 and were designed with Gene Runner Software 6.0 (https://gene-runner.software.informer.com/6.0/, accessed on 8 May 2025) (Hasting Software, Inc., Hasting, NY, USA). All the primer sets had an annealing temperature of 60 °C. The amplification conditions were as follows: 2 min at 50 °C, 10 min at 95 °C, 40 cycles of denaturation for 15 s (s), and annealing/extension at 60 °C for 10 min. The relative expression of the studied genes was calculated according to Applied Biosystems software via the comparative threshold cycle method.

### 4.10. Histological Analysis

The sections were stained with H&E where 10% formalin saline solution was used to fix the lung tissue samples. The samples were embedded in paraffin wax (5 mm thick) after dehydration in various grades of ethyl alcohol (100%, 5 min; 95%, 2 min; 80%, 2 min; 70%, 2 min), washed with xylol, and impregnated. The samples were then sliced to a thickness of 5 µm via a rotatory microtome (Leica, Cat No. RM 2125, Brompton Road, UK) and placed on glass slides. Under a light microscope, the general histological structure of the lung was examined by staining with H&E solution (G1120, Solarbio, Cat No. 26094-02, Guzhong District, Liandong U, Majuqiao, Tongzhou District, Beijing, China) for 30 min at 55 °C [71].

Collagen fibers were stained [72] via Birefringence under polarized light or digital color thresholding via the objective, semiautomated quantification Sirius Red stain (Sigma-Aldrich, Cat No. 365548, Burlington, MA, USA) method, reducing observer bias, and visualized via a Light Microscope (Primo Star, ZEISS, Shanghai, China)

Images were captured at the Histology Department, Faculty of Medicine for Girls, Al Azhar University, using an Axiocam Erc 5 s camera (ZEISS, Cat No. CAM-ERC-5S-B China).

### 4.11. Immunohistochemistry (IHC)

#### 4.11.1. iNOS and CD68

After deparaffinizing and rehydrating the lung samples, the slices were microwaved for 15 min to extract the antigens. The sections were then exposed to blocking solution for 20 min, and an iNOS-recognizing primary antibody (rat monoclonal antibody, 1:500 dilution; Brand: Novus Biologicals, Cat No. NB300-605NCL Transduction Laboratories, San Diego, California, USA) was subsequently added for an additional night at 4 °C [73]. In accordance with the manufacturer’s instructions, a CD68-recognizing primary antibody (mouse monoclonal antibody, 1:200 dilution; Cat No. ab53444 -L-CD68; Leica Biosystems, Benton La, Newcastle Ltd., UK) was subsequently added to a humidified chamber [74,75]. Later, the secondary antibody was added for 10 min. Finally, one to two drops of diaminobenzidine (DAB) were added for 10 min. Lung sections were then counterstained with Mayer’s hematoxylin, dehydrated, and cleared. A light microscope (Primo Star, Zeiss, Cat No. 415500-9024-000, inocular head, 4×/10×/40× objectives, LED light, China) was used for examination of the sections. iNOS resulted in brownish coloration of the cytoplasm of the alveolar and bronchiolar epithelial cells, whereas CD68 stained the interstitial lung macrophages with a positive brownish color.

#### 4.11.2. Morphometric Studies

A Leica DM2500 optical microscope and a Leica ICC50 W digital camera (Leica Microsystems, Cat No. 11530002, Nussloch, Germany) were used, and a computerized image analyzer (Leica Q500 MC application) was used and shown on the screen (Leica Microsystems Ltd., Cambridge, UK). To change the measurement units (pixels) generated by the image analyzer program into actual micrometer units, the data were automatically calibrated [76]. Ten unique, nonoverlapping fields from five different lung sections in each group were examined thrice to evaluate the following:

1. The interalveolar septa thickness in H&E-stained sections at 400× magnification;
2. Sirius Red-stained sections at ×200 magnification; the mean percentage area of red-stained collagen fibers/μm^2^ in the lung interstitium and around the pulmonary blood vessels [77];
3. At 400× magnification, the mean percentage of positive iNOS immunoreactivity in the sections stained with anti-iNOS antibodies was evaluated [73];
4. An estimate of the average number of alveolar macrophages in anti-CD68 immunostained sections at 400× magnification [75,78].

To minimize bias in stained samples, we randomized all staining prior to processing and assigned coded labels to blind the evaluators. Histopathological or immunohistochemical assessments were performed by two independent observers blinded to the treatment groups. Finally, if discrepancies occurred, they were resolved through consensus or by a third evaluator.

### 4.12. Statistical Analysis

The statistical software SPSS for Windows (v23.0) IBM Corporation (Armonk, NY, USA) was used for calculations.

Parametric assumptions were systematically verified via normality testing. All continuous outcome variables (e.g., fibrosis area%, cytokine levels, and gene/protein expression) were subjected to Shapiro–Wilk normality tests (α = 0.05) per group.

The results are displayed as the mean ± SD. One-way analysis of variance (ANOVA) and a Bonferroni post hoc correction were employed to identify significant differences between groups. A *p* value ˂ 0.05 was considered statistically significant.

## 5. Conclusions

In summary. This study revealed a promising outcome in which PRP improved lung damage and reduced PF compared with Dexa.

The present study provides evidence that PRP aids in treating PF and lung damage via improved histological and histomorphometric images of lung tissue. Second, there was a significant reduction in the HP level, followed by *TWIST1* downregulation.

Limitation: The current study is limited by being conducted on experimental animals. Therefore, to use PRP in clinical practice, clinical trials must support the current findings. Further studies are also warranted to optimize the PRP dose and treatment duration (as future directions).

Therefore, the beneficial antiapoptotic and antifibrotic effects of PRP suggest new directions for the development of modern treatments for AgNP-induced tissue fibrosis. Moreover, PRP therapy can be utilized in conjunction with conventional treatment regimens with or without natural products to decrease various NP side effects (recommendation and future perspectives) [79,80,81,82,83,84].

## Figures and Tables

**Table 1:** Primers for gene expression determination via qRT–PCR.

Gene	Primer Sequence
*CASP3*	Forward primer 5′-GTGGAACTGACGATGATATGGC-3′ Reverse primer 5′-CGCAAAGTGACTGGATGAACC-3′
*TWIST1*	Forward primer 5′-CCGGAGACCTAGATGTCATTGT-3′ Reverse primer 5′-CTGGGAATCTCTGTCCACCG-3′

## Data Availability

Data is contained within the article or Supplementary Materials.

## Supplementary Materials

The following supporting information can be downloaded at: https://www.mdpi.com/article/10.3390/ijms26146782/s1.

## Abbreviations

AgNPs	Silver nanoparticles
ANOVA	One-way analysis of variance
BWt	Body weight
CaCl_2_	Calcium chloride
CD68	Cluster of differentiation 68
DAB	Diaminobenzidine
DLS	Dynamic light scattering
Fcc	Face-centered cubic
H&E	Hematoxylin and eosin
HP	Hydroxyproline (HP)
IHC	Immunohistochemical
iNOS	Inducible nitric oxide synthetase
PBS	Phosphate-buffered saline
PF	Pulmonary fibrosis
PPP	Platelet-poor plasma
qRT–PCR	Quantitative real-time polymerase chain reaction
SD	Standard deviation
SEM	Scanning electron microscope
TEM	Transmission electron microscopy
XRD	X-ray diffraction
